# Host habitat shapes the core gut bacteria of decapod crustaceans: A meta-analysis

**DOI:** 10.1016/j.heliyon.2023.e16511

**Published:** 2023-05-23

**Authors:** Md Javed Foysal

**Affiliations:** Department of Genetic Engineering and Biotechnology, Shahjalal University of Science and Technology, Sylhet, 3114, Bangladesh

**Keywords:** Aquaculture, Rearing environment, Salinity, Gut microbiota, Bioinformatics

## Abstract

Gut microbiota is an essential determinant factor that drives the physiological, immunological, and metabolic functions of animals. A few meta-analysis studies identified crucial information about the gut microbiota of vertebrate animals in different habitats including fish while no report is yet available for the commercially cultured decapod crustaceans (DC). This meta-analysis investigated the gut microbiota of 11 commercially cultured DC species from five different groups-crab, crayfish, lobster, prawn, and shrimp to gain an overview of microbial diversity and composition and to find out core genera under two different host habitats: freshwater and saltwater. The analysis of 627 Illumina datasets from 25 published studies revealed selective patterns of diversity and compositional differences among groups and between freshwater and saltwater culture systems. The study found a salinity-dependent heterogeneous response of gut microbiota, specifically *Vibrio* in saltwater for white shrimp, a species that can be cultured with and without salt. Overall, the genera reared in freshwater showed higher diversity in the gut microbial communities than those reared in saltwater. An overwhelming abundance of *Candidatus* Bacilloplasma and *Vibrio* were identified for species cultured in freshwater and saltwater system, respectively and these two species were identified as the main core genera for nine out of 11 DC species, except freshwater prawn and river prawn. Together, these results demonstrate the effectiveness of the meta-analysis in identifying the robust and reproducible features of DC gut microbiota for different groups and host habitats. The diversity information curated here could be used as a reference for future studies to differentiate various DC species under two different rearing environments.

## Graphical abstract

**Figure:**
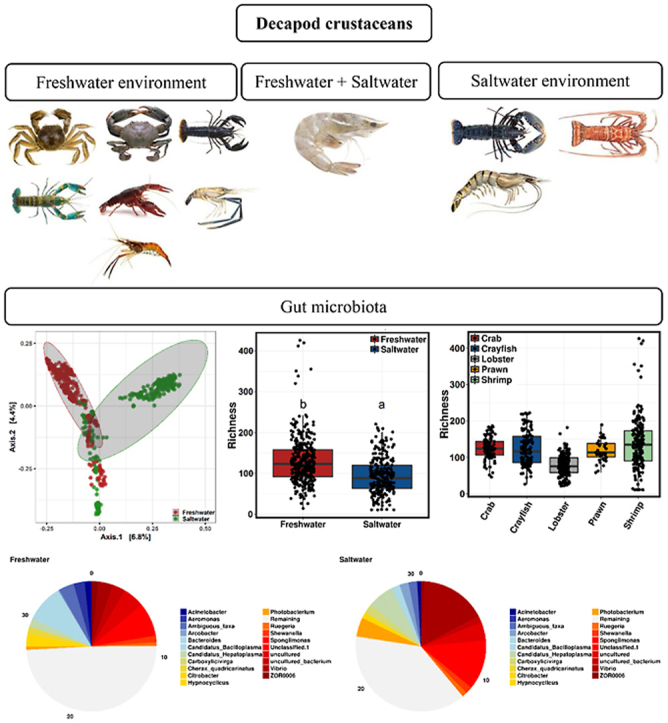

## 1 Introduction

Crustacean signifies one of the most diverse species-rich animal groups in the aquatic ecosystem with large variations in sizes, morphologies, and feeding natures. Decapoda is a subgroup of malacostracan crustaceans that includes highly-valued edible shellfish such as crabs, shrimps, prawns, lobster, and crayfish [1]. Global aquaculture of decapod crustaceans (DC) is growing with more than 7.9 million tons of production per year for human consumption [2]. They are adapted to endure significant variations in toxicity, temperature, pH, and salinity, and considering their biological characteristics and wider availability, these are considered model organisms for physiological, biochemical, and ecological research [[3], [4], [5], [6]]. Furthermore, analysis of variations in the metabolism and immunity with a biological marker could provide a new insight for future studies on the management of commercially cultured DC species in terms of growth performance, immunity and disease resistance.

The animal gut carries diverse bacterial communities that play a crucial role in producing short-chain fatty acids and vitamins, preventing colonization by pathogens, as well as in the development and conservation of immunity [[7], [8], [9]]. These properties provide the gut microbial community an attractive target for dietary interventions to improve the growth, immunity, and production of aquatic animals. With the advancement in high throughput sequencing, gut microbiota analysis has become an active and indispensable field of animal research [10,11]. An ample number of studies have been conducted on the gut microbiota of aquatic animals including commercially cultured DC species under various environmental conditions and feeding regimes, primarily by sequencing of bacterial 16 S rRNA gene. Considering the number of studies and volume of data for gut microbiota of DC, the reproducibility of microbial communities for the same species or group under different habitats or diets remains debatable. In addition, most studies have focused on the identification of beneficial or pathogenic bacteria and thus leaving the most abundant bacteria behind the shadow. Therefore, a solid overview of gut microbiota for a species has often been overlooked under the massive volume of data.

The myriad of HTS studies on gut microbiota can be combined and compared through meta-analysis which may reveal the patterns of microbial community and composition for a similar sample type. In DC, gut microbiota has been reported based on life stages [[12], [13], [14], [15]], feed additives [[16], [17], [18], [19]], host habitats [[20], [21], [22], [23]], and health status [[24], [25], [26]]. The results of these studies showed significant modulations in gut microbial communities in response to dietary treatments, diseases, life cycles and rearing water. However, despite significant variations and diversity, some of the microbes in the gut perform fundamental biological functions of the host, regardless of host factors and rearing conditions are referred to as “core” or “resident” microbiota.

The main objective of this study was to curate and re-analyse the diversity and composition of DC gut microbiota from 33 published studies to find out core gut genera irrespective of diets, host habitats and growth conditions. This was achieved by applying programmed computational pipelines across datasets with the aid of statistical power to identify reproducible signals against gut microbiota of DC species under a common factor reported in all studies.

## 2 Methods

### 2.1 Databases and inclusion criteria

The keywords “16 S”, “gut microbiota”, “crustaceans”, “shrimp”, “prawn”, “lobster”, “crayfish” were used search for the publications in Scopus and Web of Science. Additionally, commercially cultured decapod crustaceans including mitten crab, mud crab, tiger shrimp, white shrimp, freshwater prawn, river prawn, red claw, red swamp and marron were individually searched in NCBI SRA and BioProject for 16 S rRNA sequence data for gut microbiota. A total of 33 published articles (Table 1) were found wherein 32 accession numbers are publicly available. The unpublished data of white shrimp gut microbiota study by Elizondo-González et al. [17] was collected from corresponding author on request. Present study included all metadata assigned to each Bio-sample assigned in the database. However, information like sex, weight, life stages, diets and health status are not available for all bio-samples and published articles except species names and rearing water. Therefore, species and groups (crab, shrimp, prawn, crayfish) were considered for further analysis. Ocean and sea water were categorized as salt water whereas underground freshwater, pond, lake and river water were considered as freshwater. *Macrobrachium nipponense* commonly known as oriental river prawn has been named “river prawn” and *M. rosenbergii* also known as giant freshwater prawn has been termed as “freshwater prawn” in this study.

**Table 1:** Studies and databases reporting gut microbiota in decapod crustaceans.

DC	Environment	Year	Accession	Samples	Technology	Reference
Tiger shrimp	Saltwater	2016	SRP059721	3	Illumina	[25]
Tiger shrimp	Saltwater	2020	PRJNA540737	9	Illumina	[77]
Tiger shrimp	Saltwater	2020	PRJNA553862	30	Illumina	[78]
FW prawn^‡^	Freshwater	2016	SRR1502207	1	Roche 454	[66]
River prawn	Freshwater	2015	PRJNA280489	6	Roche 454	[21]
River prawn	Freshwater	2017	PRJNA354668	17	Illumina	[67]
River prawn	Freshwater	2018	PRJNA381860	64	Illumina	[22]
White shrimp	Freshwater	2020	Provided	8	Illumina	[17]
White shrimp	Saltwater	2020	PRJNA600113	6	Illumina	[16]
White shrimp	Freshwater	2018	PRJNA422950	15	Illumina	[23]
White shrimp	Freshwater	2019	SRP128484	15	Illumina	[19]
White shrimp	Saltwater	2019	SRP136220	7	Illumina	[12]
White shrimp	Saltwater	2020	PRJNA578594	9	Illumina	[26]
White shrimp	Freshwater	2019	PRJNA505962	6	Illumina	[79]
White shrimp	Saltwater	2017	SRX2946975	1	Illumina	[14]
White shrimp	Freshwater	2019	PRJNA522274	44	Illumina	[80]
White shrimp	Saltwater	2018	PRJNA352369	14	Ion-Torrent	[81]
EU lobster^‡^	Saltwater	2019	PRJNA577421	161	Illumina	[20]
Spiny lobster	Saltwater	2017	PRJNA396648	18	Roche 454	[13]
Mitten crab	Freshwater	2018	SRP110849	8	Illumina	[82]
Mitten crab	Freshwater	2020	PRJNA646327	18	Ion-Torrent	[83]
Mitten crab	Freshwater	2020	PRJNA530094	43	Illumina	[84]
Mud crab	Saltwater	2020	SRP215842	24	Illumina	[85]
Red-Swamp^†^	Freshwater	2020	PRJNA609648	26	Illumina	[15]
Red-Swamp^†^	Freshwater	2020	PRJNA557576	5	Illumina	[72]
Red-Claws^†^	Freshwater	2019	PRJNA573062	6	Illumina	Unpublished
Red-Claws^†^	Freshwater	2020	PRJNA634222	16	Ion-Torrent	[24]
Marron^†^	Freshwater	2020	PRJNA505066	12	Illumina	[86]
Marron^†^	Freshwater	2020	PRJNA579035	12	Illumina	[18]
Marron^†^	Freshwater	2020	PRJNA549032	16	Illumina	[87]
Marron^†^	Freshwater	2020	PRJNA549030	8	Illumina	[88]
Marron^†^	Freshwater	2021	PRJNA609769	12	Illumina	[76]
Marron^†^	Freshwater	2021	PRJNA682157	42	Illumina	[89]

FW prawn^‡^, Freshwater prawn, EU lobster^‡^, European lobster. ^†^Crayfish.

### 2.2 Data curation and validation

The publicly available 682 gut microbial samples from 33 studies (Table 1) were downloaded from NCBI databases using SRA Tookit [27,28]. Ten articles for white shrimp, six for marron, three for each tiger shrimp, river prawn, mitten crab, two for red claw and red swamp, and one for freshwater prawn, mud crab, European lobster and spiny lobster were used for sequence data curation in this study. Paired-end (PE) and single end (SE) sequences were stored separately. For PE sequencing, forward and reverse sequences were separated using fastq-dump split-files. Phred score Q30 (error rate 1 in 1000) was used for the selection of high-quality reads that removed all Ion-Torrent and Roche sequence data (except for spiny lobster) and one Illumina data. A total of 627 samples from 27 studies were retained after primary screening. Additionally, two studies with small sample sizes (n ≤ 5) that failed to generate sufficient power scores (<0.80) were excluded and 623 samples were considered for further analysis. To maintain homogeneity in meta-analysis, two different hypervariable regions – V1V3 and V3V4 were checked further for any differences in diversity estimates after classification (Fig. S1).

### 2.3 Processing of sequence and metadata

**Fig. 1:**
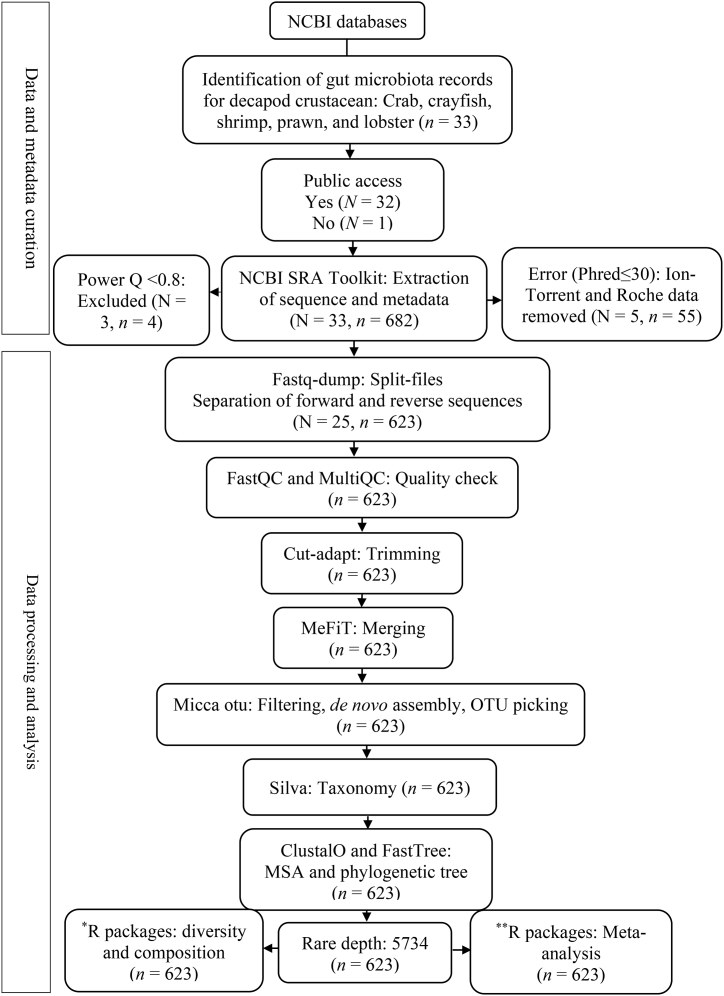
Flowchart representing steps and methodologies for the meta-analysis of decapod crustacean gut microbiome data. N = Number of studies, n = number of samples.

Illumina primers and adapter sequences were trimmed only with Cutadapt to retain maximum biological information [29]. The merging and filtering tool, MeFiT was used for the joining of overlapping paired-end reads [30]. FastQC [31] and MICCA stats [32] were used to check the quality of raw and processed reads. Micca OTUs (v1.7.0) used for the filtering of merged reads, cleaning of chimeric reads and singleton, picking and hybrid *de novo* greedy clustering of Operational Taxonomic Units (OTUs) at 97% similarity index [32,33]. Taxonomic classification of the representative OTUs was performed at 97% resemblance against SILVA 1.32 release [34] at 0.5 confidence threshold level. For multiple sequence alignment, clustalO program [35] was employed followed by phylogenetic tree constructions under GTR + CAT model in FastTree [36]. Unannotated OTUs were labeled as unclassified whereas classified OTUs for uncultured and ambiguous taxa were renamed as “other”. Each sample was set to an even rarefied depth of 5734 for the subsequent measurements of alpha-beta diversity and microbial composition in QIIME (v1.9.1) and Rstudio (Fig. 1).

### 2.4 Downstream bioinformatics

For the assessment of sequence depth and coverage, iNEXT R package [37] was used to visualize the depth of species diversity in the rarefied data. Alpha diversity was calculated in terms of richness, Shannon, Simpson and chao1 diversity using rarefied OTUs abundance in phyloseq [38]. Beta-ordination in term of Principal Coordinate Analysis (PCoA) was analysed with microbiome (https://microbiome.github.io/tutorials/), vegan [39], microbiomeSeq (https://github.com/umerijaz/microbiomeSeq) and phyloseq R packages. The relative abundance of bacteria at genus level was calculated in ampvis2 [40] and phyloseq R packages. Here, the analysis was based on induvial crustacean species, groups and types of rearing water.

### 2.5 Statistical analysis

Data analysis were performed in R statistical packages (v4.1.1) [41]. R package “pawr” was used to calculate power score in meta-analysis [42]. Meta-analysis performed in “ape” [43], “dendextend” [44], “meta” [45], “hilldiv” [46], “dmetar” [47], “metaviz” [48], “microbiome” (https://github.com/microbiome/microbiome), “phytools” [49], “phylosignal” [50], “treedist” (https://github.com/ms609/TreeDist), and “vegan” [39] R packages. One-way ANOVA with non-parametric Kruskal-Wallis and Dunn's tests for multiple comparison were used to compare alpha diversity measurements among decapod crustacean species. Each dataset of sequence was subjected to multiple hypothesis testing using “Bonferroni” correction to avoid false-discovery rate. Man-Whitney *U* test followed by “Bonferroni” correction used to compare diversity of gut microbiota between freshwater and saltwater environment. Beta-ordination was performed based on Bray-Curtis dissimilarity of weighted and unweighted UniFrac matric, and the dispersion differences of centroid was measured using permutational multivariate analysis of variance (PERMANOVA). Generalized Adaptive Model for environment as implemented in metamicrobiomeR: *taxa. compare* to analyse taxonomic differences between species reared in freshwater and saltwater. Then random effect meta-analysis model applied in metamicrobiomeR: *meta. taxa* to estimate standard errors, overall effects and heterogeneity across different species under two different rearing water. The object class of meta in terms of differences in richness at genus level between freshwater and saltwater reared species was visualized as forest plot. To avoid biasness, minimum prevalence of ≥2% reads at genus level was considered for meta-analysis of microbial taxa. *p*-value of <0.05 was considered as statistically significant in all stages of data analysis. Fisher presence-absence test and core function test in “microbiome” R package were performed to identify core gut microbiome in individual species. At least ≥2% of read abundance in more than 95% of samples for a species was classified as “core” gut microbiota.

## 3 Results

### 3.1 Study selection and sequence statistics

After initial screening and evaluation of 46 studies, the 16 S rRNA sequences and metadata were retrieved from 33 publicly available datasets that met the inclusion criteria as described in methods. To increase comparability among studies, a common and uniform bioinformatic pipeline was developed to deal with heterogeneous data and re-generate information about relative abundance. To minimize bias, sequence data were aggregated to curate up to genus-level information targeting 16 S rRNA gene. A total of 44.5 million reads, with an average of 70,084 ± 9314.8 reads per sample were obtained from 25 studies and 627 samples that were classified into 36,371 OTUs, 8 phyla, and 1751 genera. Despite some ascending lines, the rarefaction curve showed that most of the samples captured major diversity, up to the saturation level (Fig. S2). Alpha-beta diversity, and hill numbers based on the richness (the mean number of taxa detected) were used to analyse changes in microbial communities.

### 3.2 Microbial diversity in specific groups and host habitats

**Fig. 2:**
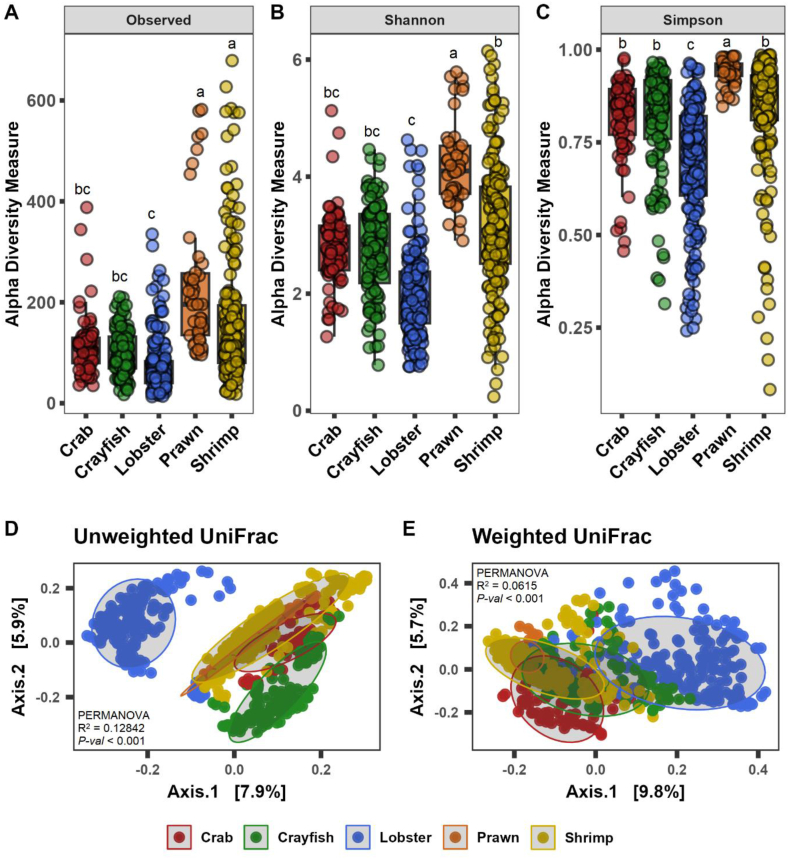
Alpha-beta diversity in five major decapod crustacean groups (N = 25) – crab (n = 75), crayfish (n = 139), lobster (n = 179), prawn (n = 81) and shrimp (n = 149). (A–C) Alpha diversity measurements in terms of observed species, Shannon, and Simpson. (D–E) Beta-ordination in terms of unweighted (presence-absence) and weighted (relative abundance) UniFrac metric. N = Number of studies, n = number of samples.

**Fig. 3:**
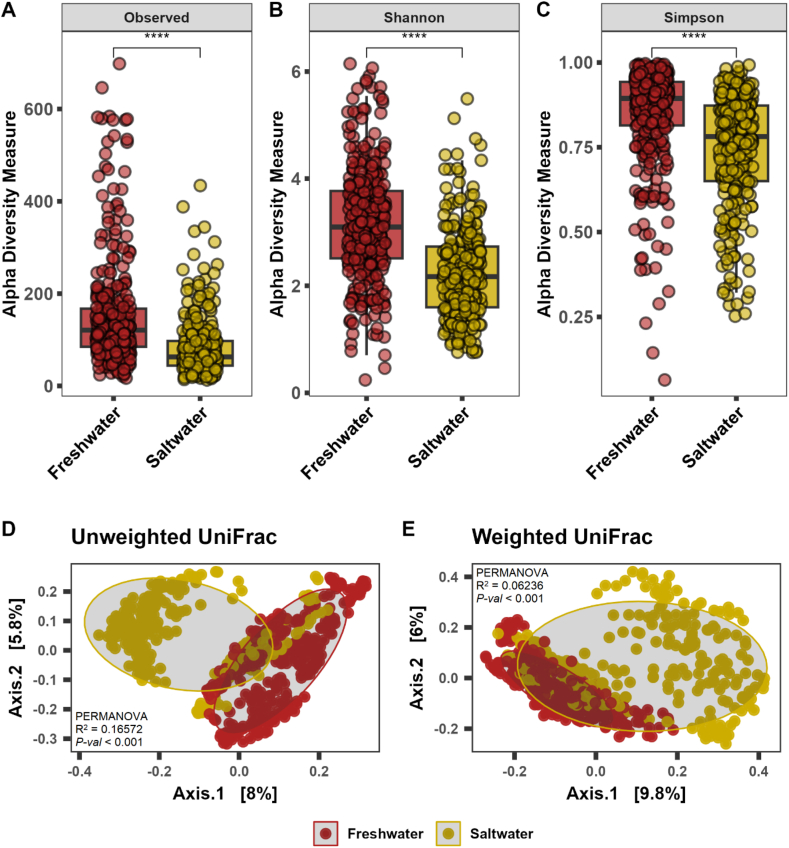
Alpha-beta diversity in decapod crustacean gut based on rearing water (N = 25) – freshwater (n = 359) and saltwater (n = 264). (A–C) Alpha diversity measurements in terms of observed species, Shannon, and Simpson. (D–E) Beta-ordination in terms of unweighted (presence-absence) and weighted (relative abundance) UniFrac matric. N = Number of studies, n = number of samples.

**Fig. 4:**
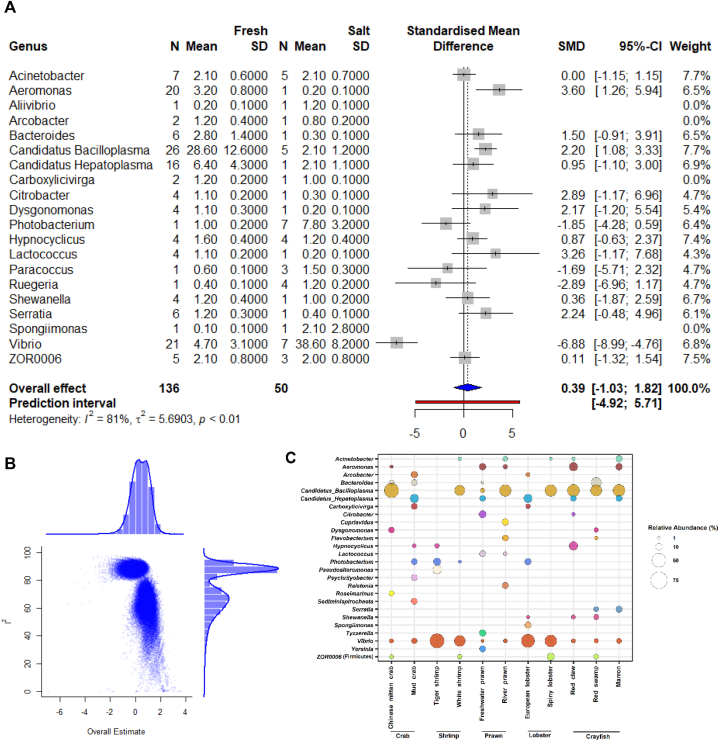
Bacterial composition in the gut of major decapod crustacean (N = 25). (A) Forrest plot showing the compositional differences of gut microbiota between two different host habitats – freshwater (n = 359) and saltwater (n = 264). The adjusted lines and confidence interval (95% – CI) were generated from generalized adaptive mixed model (GAMM). (B) Overall estimates of heterogeneity (I^2^) in the decapod crustacean's gut microbiota based on rearing water. (C) Relative abundance of bacteria at genus level in 11 different decapod crustacean species – mitten crab (n = 51), European lobster (n = 161), freshwater shrimp (n = 17), marron (n = 102), mud crab (n = 24), red-claw (n = 6), red-swamp (n = 31), river prawn (n = 64), spiny lobster (n = 18), tiger shrimp (n = 39), and white shrimp (n = 110). Genera with at least 5% annotated read abundance in any of the species are presented here.

The Shannon estimates and beta-ordination showed no significant differences in the abundance and diversity of microbial communities across curated datasets for different hypervariable regions (Fig. S1). The overall meta-analysis of alpha diversity showed increased species richness (observed and Chao1) in shrimp and prawn; however, prawn gut microbiota found more even (Shannon and Simpson), in relation to other species (Fig. 2A–C). The beta-ordination unweighted (PERMANOVA R = 0.1284, *p*-value <0.001) and weighted (PERMANOVA R = 0.0615, *p*-value <0.001) UniFrac distance metric showed distinct differences of gut microbiota between species (Figure D–E). The ordination and centroid analysis of beta-dispersion also showed that crustacean species tended to exhibit more differences in gut microbiota when measuring the presence of rare taxa in the communities (Tables S1 and S2). Next, diversity meta-analysis was performed based on type of rearing water, freshwater and salt water. The results indicated increased diversity and evenness of gut bacterial communities in species reared with freshwater compared to saltwater (Fig. 3A–C). Discrete clustering of bacteria (unweighted PERMANOVA R = 0.1657, p-value <0.001; weighted PERMANOVA R = 0.06236, p-value<0.001) (Fig. 3D–E), high heterogeneity (I^2^ = 81.0%, τ^2^ = 5.6903, p-value <0.01) suggest that the differences of gut microbial communities are more pronounced under type of rearing water than the host species itself (Fig. 4A–B).

### 3.3 Microbial composition in specific groups and host habitats

**Fig. 5:**
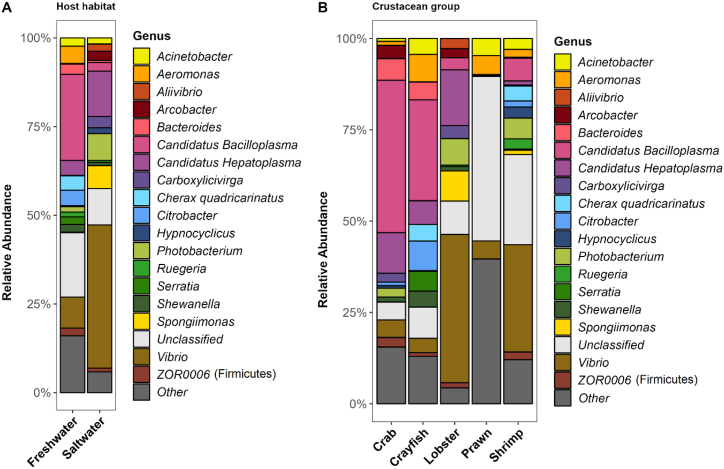
Gut microbiota of major decapod crustacean based on published articles (N = 25). (A) Relative abundance of bacteria at genus level in two different host habitats – freshwater (n = 359) and saltwater (n = 264). (B) Relative abundance of bacteria at genus level in five different crustacean groups – crab (n = 75), crayfish (n = 139), lobster (n = 179), prawn (n = 81) and shrimp (n = 149). Un-assigned OTUs are presented as unclassified while bacteria with less than <2% abundance are grouped as “other”. N = Number of studies, n = number of samples.

**Fig. 6:**
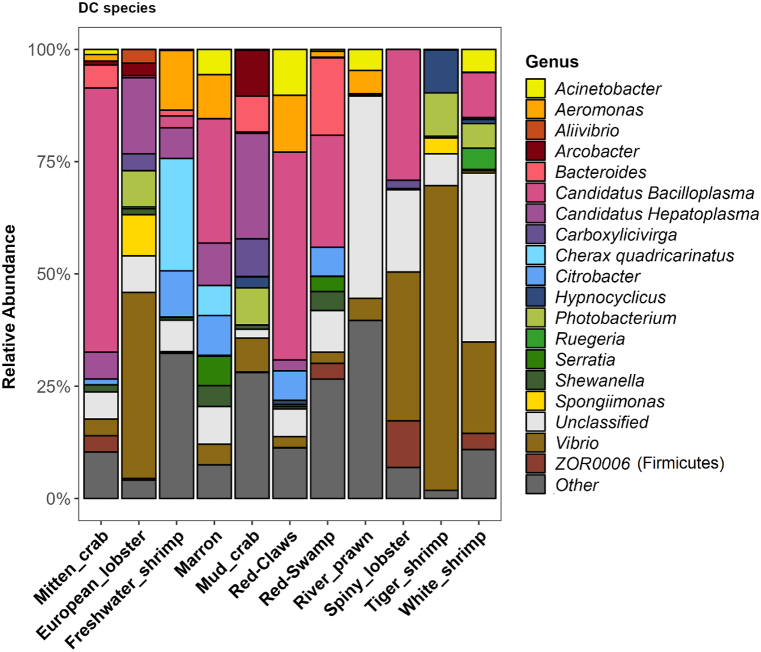
Relative abundance of bacteria at genus level in 11 major decapod crustacean species based on published articles (N = 25). The crustacean species are mitten crab (n = 51), European lobster (n = 161), freshwater shrimp (n = 17), marron (n = 102), mud crab (n = 24), red-claw (n = 6), red-swamp (n = 31), river prawn (n = 64), spiny lobster (n = 18), tiger shrimp (n = 39), and white shrimp (n = 110). Un-assigned OTUs are presented as unclassified while bacteria with less than <2% abundance are grouped as “other”. N = Number of studies, n = number of samples.

As 62 bacterial genera had more than 1% of read abundance in at least one crustacean species, a higher abundance cut-off value (≥2%) was considered for diversity-based meta-analysis of gut microbiota using Generalized Adaptive Models for Location, Scale and Shape (GAMLSS). From the classified sequences, 20 genera contributed 46% of the abundance wherein freshwater harboured more bacteria than saltwater. *Photobacterium*, *Paracoccus*, *Ruegeria*, and *Vibrio* were found in higher mean abundance (SMD) in saltwater than freshwater (Fig. 4A and C, Fig. 5A, Fig. S3). However, *Vibrio* seems to be more heavily influenced by the presence of salt in the rearing water (Fig. 4A, Fig. S3). Comparatively, more species including *Aeromonas*, *Candidatus* Bacilloplasma, *Ca.* Hepatoplasma, *Citrobacter*, *Dysgonomonas*, *Lactococcus*, and *Serratia* were positively influenced in the gut communities without salt in the rearing environment (Fig. 4A). Though *Vibrio* has been identified in all species regardless of rearing water; however, the abundance was found below 5% under freshwater, compared to >25% in saltwater and showed significant (*p*-value<0.05) log-fold enrichments (Figs. S3–5). Genera from *Candidatus* lineages predominantly favour freshwater and found in low abundance for the European lobster and tiger shrimp in saltwater. However, higher abundance of *Ca*. Bacilloplasma in spiey lobster and *Ca*. Hepatoplasma in European lobster suggest the association of some other host intrinsic factors in favouring the growth of a species from a genera or lineage. Additionally, the results of log-fold changes (*p*-value = 0.0821) suggest that this bacterium cannot be used as a biological marker to differentiate gut microbiota between salt and freshwater like *Vibrio*. The genus *Aeromonas* although showed enrichment in freshwater culture system; however, it could not generate the significant log-fold threshold due to very low abundance (<0.2%) of *Aeromonas* in six out of 11 species (Fig. 4C). Surprisingly, majority of OTUs generated for prawn remained unclassified while other had very low abundance (<2%) (Fig. 5B and 6). Interestingly, overwhelming abundance (>50%) of *Ca*. Bacilloplasma in red claws and mitten crab, and *Vibrio* in tiger shrimp was observed (Fig. 6). In this study, though 116 OTUs were assigned to archaeal kingdom, none of the genus had >2% read abundance in any sample. Archaea was present in lobster (0.09%), shrimp (0.06%), crayfish (0.01)) and crab (0.002%) but not in prawn. The decapod species under saltwater environment had twice read abundance for archaea (0.1%) than freshwater (0.05%). All sequences of archaea for carb under saltwater were assigned to *Methanoculleus* (0.02%) that was absent for same species under freshwater culture system. *Methanobrevibacter* (0.01%) was the only archaea present in the crayfish gut under freshwater culture system. *Methanosaeta* was predominant archaea in shrimp gut in both host habitats. The most abundant archaea *Candidatus* Nitrosopumilus was only present in the Lobster gut (Fig. S5).

### 3.4 Core gut microbiota in decapod crustaceans

To gain further insights into microbial compositional differences, the microbiota for 11 individual species at genus level was analysed to find core or resident gut bacteria. *Vibrio* was the only genus identified in more than 95% of samples using Fisher presence-absence test. Next to *Vibrio*, *Ca*. Bacilloplasma and *Aeromonas* were identified from 78.4% to 67.5% of the samples (*n* = 627). The core analysis of dataset in “microbiome” R also supports Fisher test where only *Vibrio* showed 95.5% relative population frequencies at 1% compositional abundance threshold. Finally, Fisher test was applied to find core genera in individual decapod crustacean species regardless of culture environment. *Ca*. Bacilloplasma (>95%) was identified as core genera in five species namely Chinese mitten crab, marron, red claw, red swamp and white shrimp. The percentage for *Ca*. Bacilloplasma enhanced up to 98.6% across all samples for crayfish-marron, red claw and red swamp (Table S3). Four species, mainly shrimp (tiger and white shrimp) and lobster (European and spiny lobster) hosted Vibrio as core gut genera. Except *Vibrio* and *Ca.* Bacilloplasma, freshwater prawn and river prawn anchored *Aeromonas* (90.5%) and *Cupriavidus* (89.7%) as core gut genera (Fig. S6).

## 4 Discussion

With the passage of time, studies demonstrating gut microbial communities in decapod crustaceans under different culture conditions, feeding regimes, health and immune status has gained significant momentum. Meta-analysis of gut microbiota has been considered as the most potential method to study consistency, reliability and reproducibility among the studies [51]. Additionally, analysis of large-scale dataset allows to monitor dysbiosis of gut microbiota by studying most prevalent and dominant bacteria, referred as core or resident bacteria in animals [52]. This type of analysis is potentially crucial to monitor growth performance, health and immunity, disease susceptibility, and energy homeostasis of aquatic animals [53,54]. Profiling of comprehensive core microbiota for a species can be used as a model to define healthy gut microbiota and guiding their intervention to host health [55]. In this meta-analysis, data was curated from 33 studies to create baseline information about the structure and composition of decapod crustaceans’ gut microbiota including core microbiota under fresh and saltwater culture systems. With this meta-analysis, this study identified two major features of decapod crustacean gut microbiota: (i) gut microbial communities are rich in freshwater culture system; (ii) the rearing environment or host habitat plays a crucial role in defining core gut microbiota.

The results of this study showed a more influential effect of rearing environment for aquatic species [[56], [57], [58]] than culture species or diet [59]. Unfortunately, published reports on host habitats as a driver of animal gut microbiota [57,60] have no in-depth information at lower taxa levels (i.e., genus, species). Therefore, the indicator genera to define a specific host habitat remained unidentified for decapod crustaceans. An in-depth analysis of white shrimp gut microbiota in this study showed that the host favoured the colonization of *Vibrio* in saltwater and *Ca.* Bacilloplasma in freshwater (Fig. S2). These results however do not reflect the findings of Dulski et al. [61] and Rudi et al. [60] studies which reported little or no effects of salinity on pike fry (*Esox lucius*) and higher microbial diversity in the gut of Atlantic salmon (*Salmo salar*) under the saltwater system, respectively. The concentration of salt might play a significant role in selecting halophilic bacteria in the crustacean gut, preventing colonization of non-salt-loving bacteria. Therefore, despite the variations in gut microbial diversity for the same aquatic species in different published reports under identical rearing environments, the role of host habitats in shaping the gut microbiota of decapod crustaceans can be established from the present meta-analysis.

This study identified a group-specific gut microbiota in decapod crustaceans. Crayfish including marron, red claw and red swamp are freshwater species whereas lobster (European lobster and spiny lobster) grows only in saltwater. Further, white shrimp and Chinese mitten crab can grow in both environments where publish reports are only available under freshwater culture for the mitten crab. Consistent with previous findings [62], lower diversity and species richness were identified in the gut of crustacean species reared under saltwater systems to freshwater. Therefore, as a salt-dependent species, lobster showed the lowest gut microbial diversity, compared to other species. In addition, land-based cultured groups that showed low species diversity compared to sea-based cultures might also be associated with species reduction [20]. Our analysis also found that gut microbial communities are more different in terms of presence of rare taxa (unweighted) than relative abundance of classified taxa (weighted) and the findings are true and reproducible for both gut microbiota among groups (crab, crayfish, lobster, shrimp and prawn) and host habitats (freshwater-saltwater). The results suggest that certain groups of decapod crustaceans carrying specific gut microbial communities as well as host habitats favour the growth of some selective bacteria to drive definite host functions. For instance, the halophilic bacteria *Ruegeria* prevents the growth of *Vibrio* and other potential pathogens of crustaceans in high salt concentrations [63]. Therefore, an overwhelming abundance of these three bacteria can be envisaged in the fish gut under the saltwater system. Nevertheless, *Vibrio* was identified (≥1%) in all species regardless of host habitat and suggested to play some common essential host functions for decapod crustacean species. Though archaeal sequences were found in low abundance among total communities, but they are significant in terms of presence-absence, specifically those linked to nitrogen fixation including methanogenic archaea and *Ca.* Nitrosopumilus [64,65]. Further research could be directed to identify the correlations between archaea and different host habitats for decapod crustaceans.

Freshwater crayfish and crabs shared a similar core (*Ca*. Bacolliplasma) in the gut environment while freshwater prawn (*Cupriavidus*) and river prawn (*Aeromonas*, *Citrobacter*) have a different core. Currently, only four reports are available on the gut microbiota of prawns, one on freshwater prawn [66] and three on river prawn [21,67,68] that reported a low abundance of *Ca*. Bacilloplasma. Therefore, it is too early to define the core gut bacteria of freshwater prawn and river prawn. Contrarily, *Vibrio* with read abundance of more than 40% for shrimp and lobster in 14 published studies, is certainly the main core of shrimp and lobster. However, *Vibrio* together with *Aeromonas* and *Flavobacterium* is linked to several diseases of crustaceans including deadly vibriosis [69,70]. Considering higher *Vibrio* abundance and lower taxonomic resolution of amplicon sequencing at species level, it is important to identify the *Vibrio* species and their role in the crustacean's gut. Interestingly, the second core *Pseudoalteromonas* and *Ca.* Bacilloplasma showed significant differences and can be used as an indicator genus to differentiate tiger and white shrimp gut microbiota. Similarly, ZR0006, a Firmicutes (12.4% in spiny lobster, 0.6% in European lobster) differentiates the gut microbiota of two lobster species. *Candidatus* are novel lineages of Mollicutes, abundant only in the gut of crustaceans [18,20,[71], [72], [73]] and other invertebrate species [74,75] while very rare in vertebrate animals. As a core gut microbiota, they play a positive role in the health and immunity of marron, a freshwater crayfish species native to Western Australia [18,76]. Therefore, *Ca.* Bacilloplasma as core gut genus in most of the decapod crustacean species can be envisaged.

To sum up, our meta-analysis suggests large variations in the structure and composition of gut microbial communities among commercially cultured decapod crustaceans. The investigation reveals higher species abundance in freshwater culture systems with the dominance of *Vibrio* species under saltwater while *Ca.* Bacilloplasma under freshwater culture system. Furthermore, this study showed significant differences in gut microbiota in a species (white shrimp) between freshwater and saltwater culture systems. The taxonomic information generated here can be used to define healthy gut microbiota of decapod crustaceans and future reference study toward modulation of gut microbiota through diets and to monitor gut health under different host habitats. The data strongly suggest that the abundance, diversity and preservation of decapod crustacean gut microbiota is a complex process that is driven by host habitats, specifically the salinity in water.

## Author contribution statement

Md Javed Foysal: *Conceived and designed the experiment; Analysed and interpreted the data; Wrote the paper.*

## Funding statement

No fund was obtained to perform this study.

## Data availability statement

The source code used for data processing, graphic visualization and statistical analysis in this study is available on https://github.com/FoysalRon/DC-gut-microbiota.

## Declaration of competing interest

The authors declare that they have no known competing financial interests or personal relationships that could have appeared to influence the work reported in this paper.
