# Supplementary material for: Host habitat shapes the core gut bacteria of decapod crustaceans: A meta-analysis
Source: Heliyon. 2023 May 23;9(6):e16511. doi: 10.1016/j.heliyon.2023.e16511 (PMC10238905; doi:10.1016/j.heliyon.2023.e16511)
Supplement: Multimedia component 1 [file mmc1.docx]

**Supplementary data**

**Host habitat shapes the core gut bacteria of decapod crustaceans: A meta-analysis**

Md Javed Foysal ^a*^

^a^ Department of Genetic Engineering and Biotechnology, Shahjalal University of Science and Technology, Sylhet, Bangladesh

^*^Corresponding author:

Md Javed Foysal

Department of Genetic Engineering and Biotechnology

Shahjalal University of Science and Technology, Sylhet 3114, Bangladesh

Email address: mjfoysal-geb@sust.edu or javed.foysal@gmail.com

Telephone: +88 821 713491

Mobile +8801717389379

ORCID: https://orcid.org/0000-0002-2064-8897


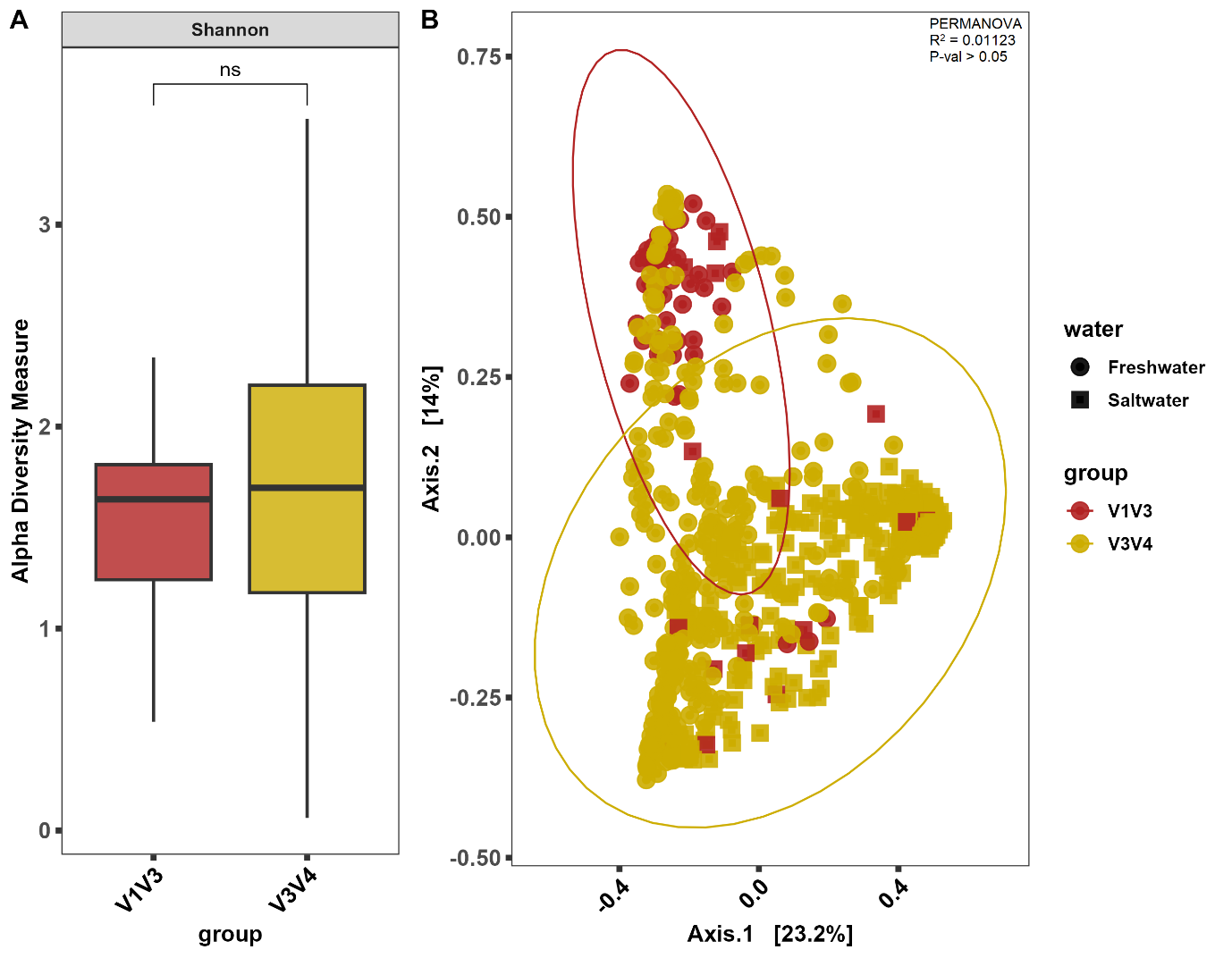


**Figure S1**. Alpha-beta diversity of crustacean gut microbiota in studies with two different hypervariable regions.


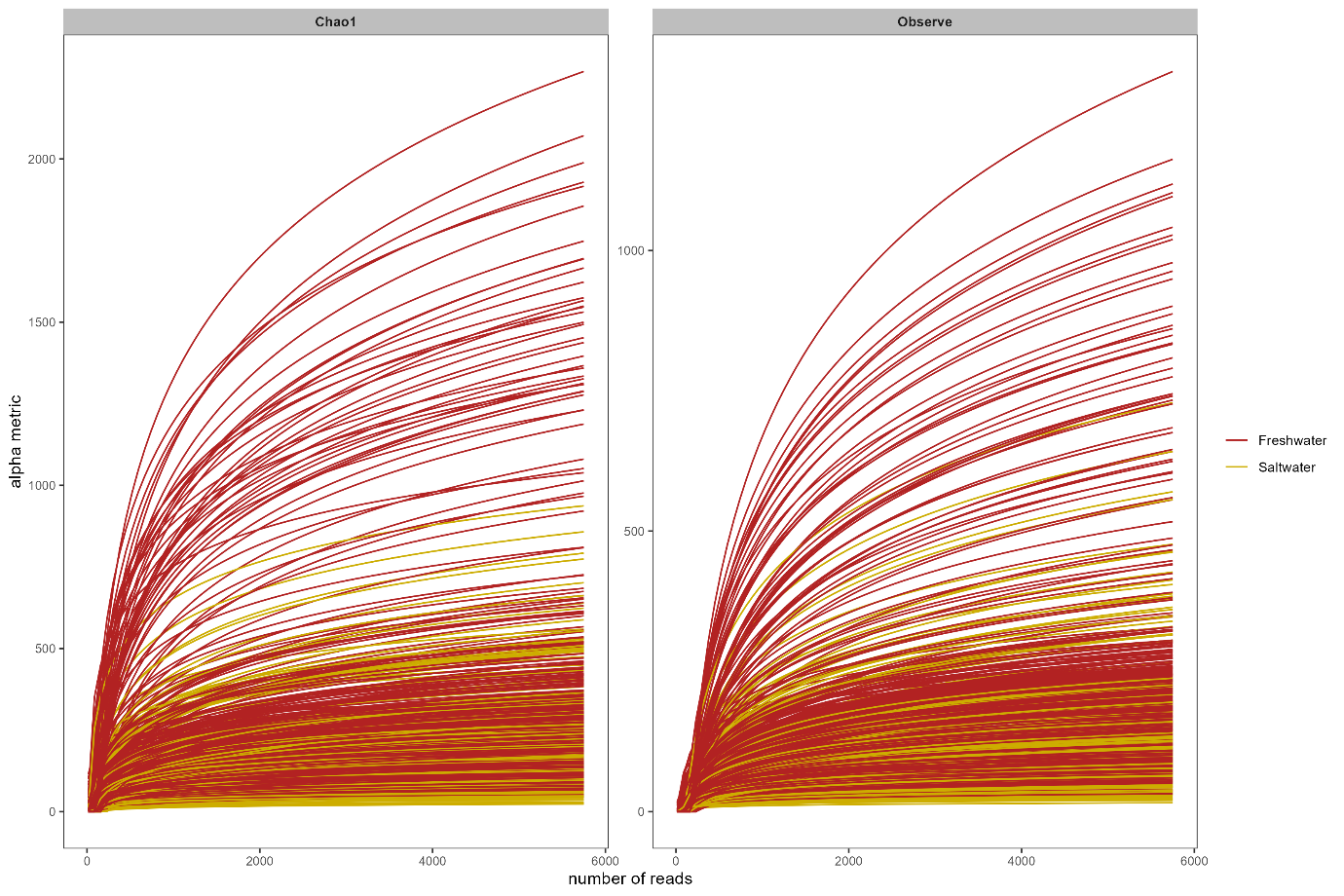


**Figure S2**. Rare-faction curve showing the depth and coverage of Illumina sequences from 627 samples.


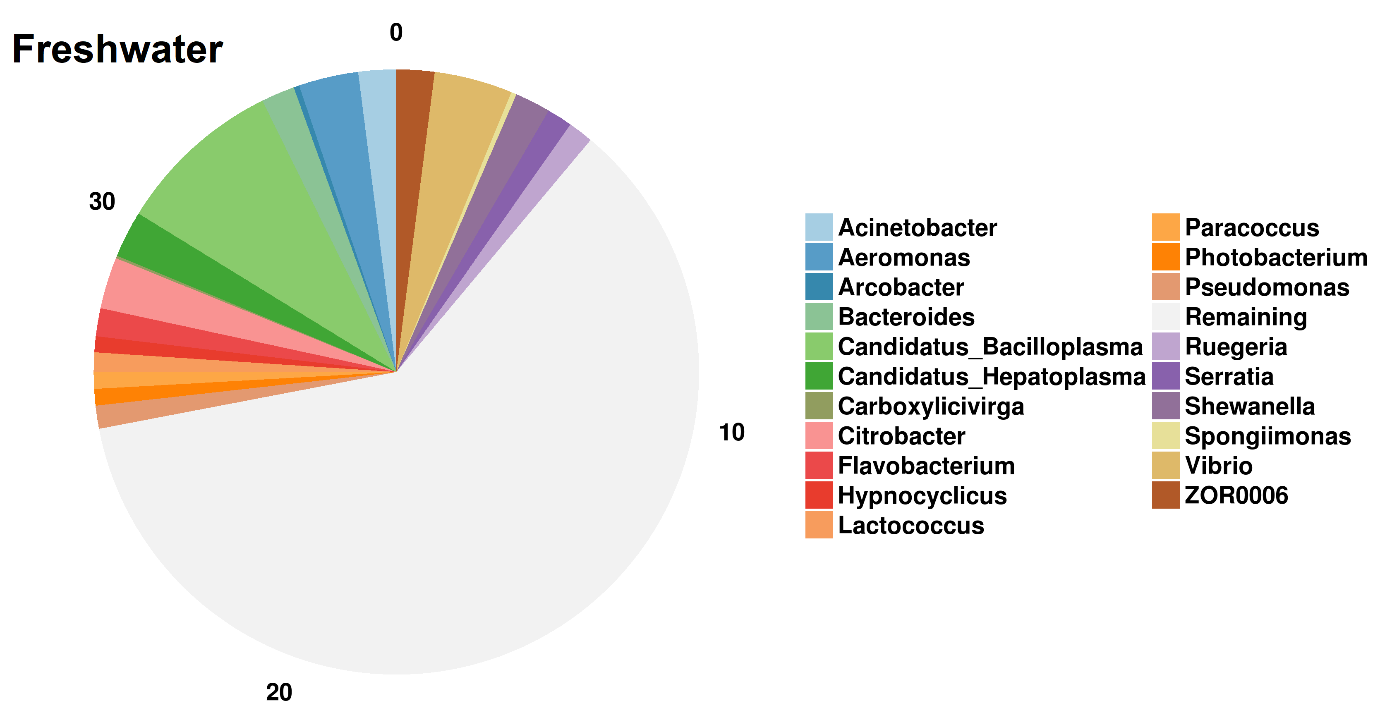

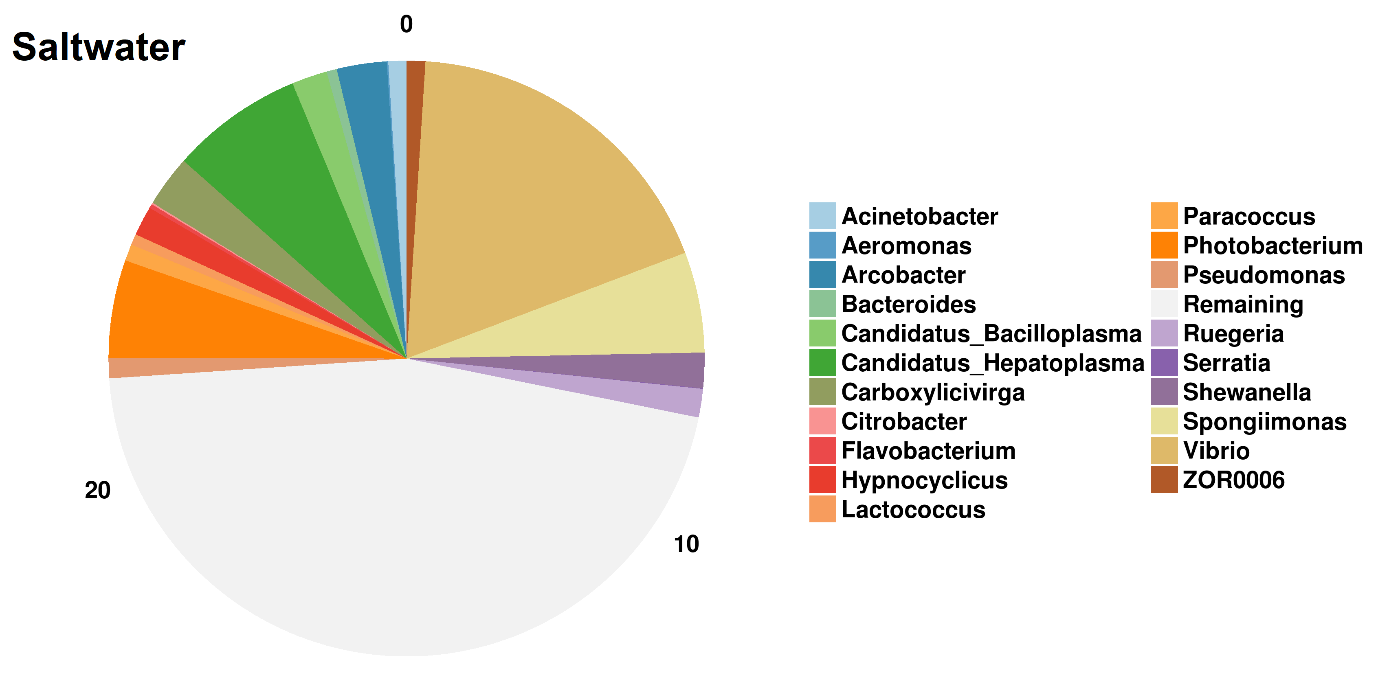


**Figure S3**. Gut microbial diversity in decapod crustacean species in two different host habitats- fresh and saltwater.


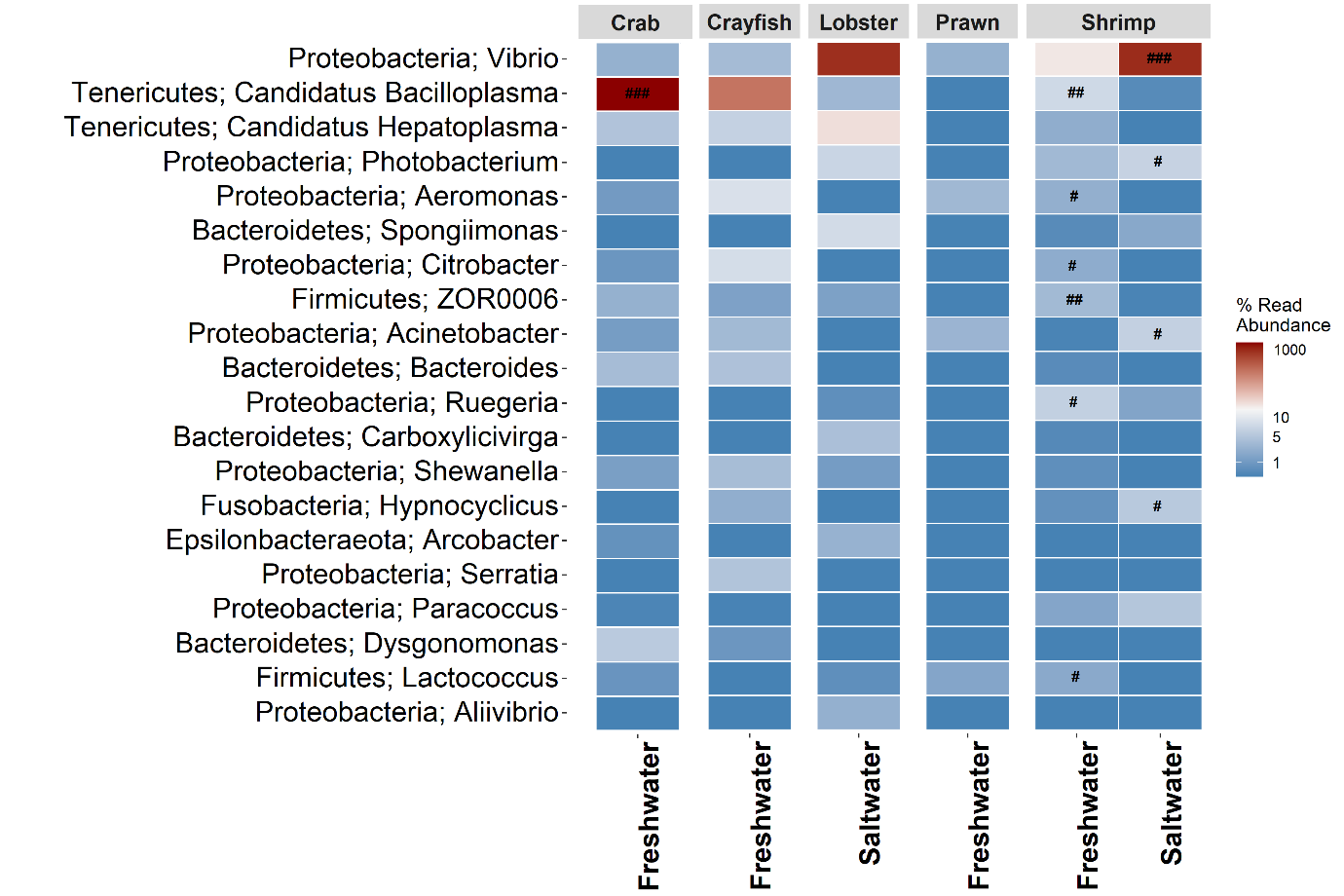


**Figure S4**. Gut microbial diversity in five groups (crab, crayfish, lobster, prawn, and shrimp) of decapod crustacean species in two different host habitats- fresh and saltwater. Log-fold changes are expressed with a different asterisk (#) in the plot. #Significantly different at α-level of 0.05. ##Significantly different at α-level of 0.005. ###Significantly different at α-level of 0.001.


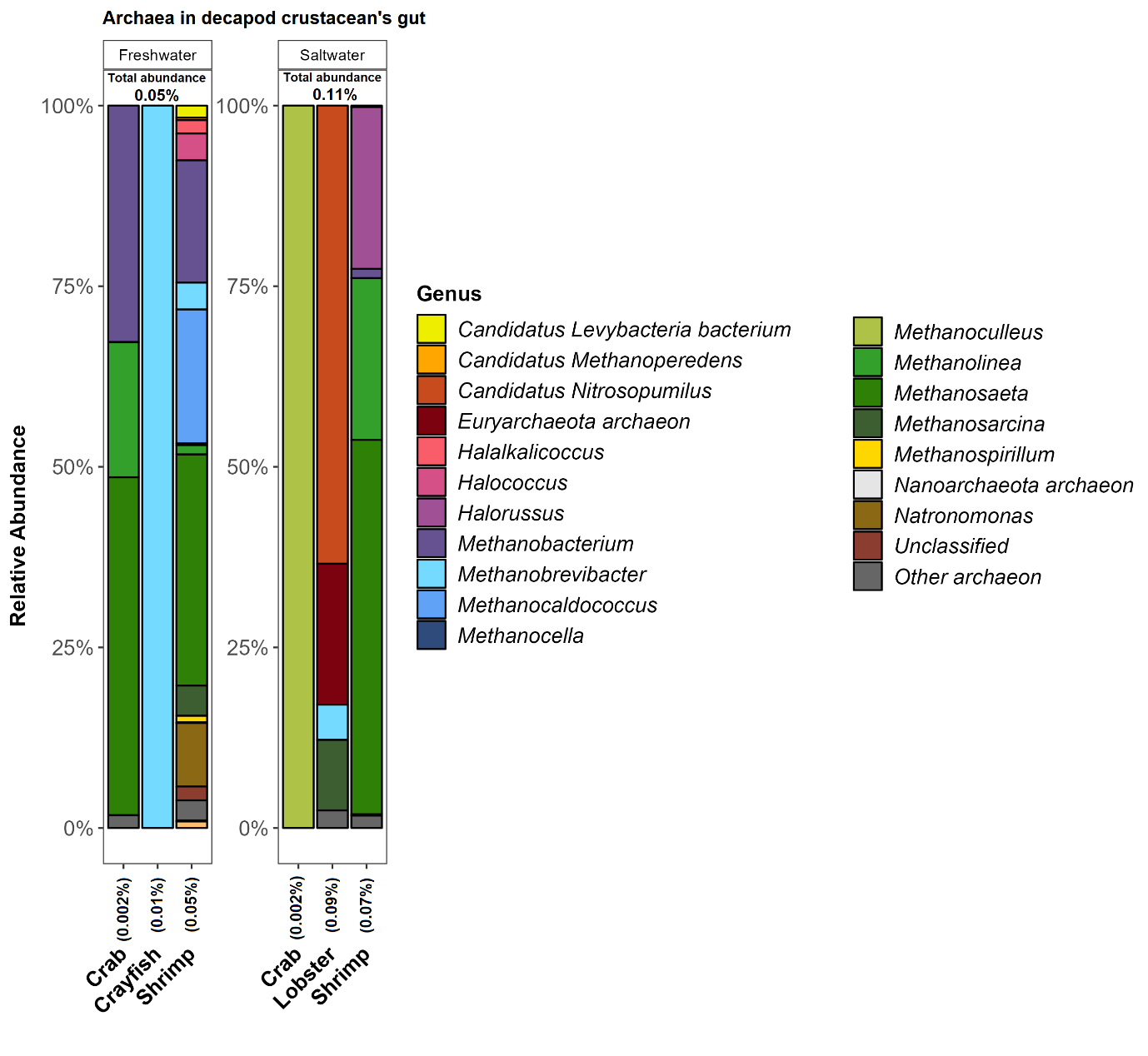


**Figure S5**. Archaeal communities in the decapod crustacean’s gut.


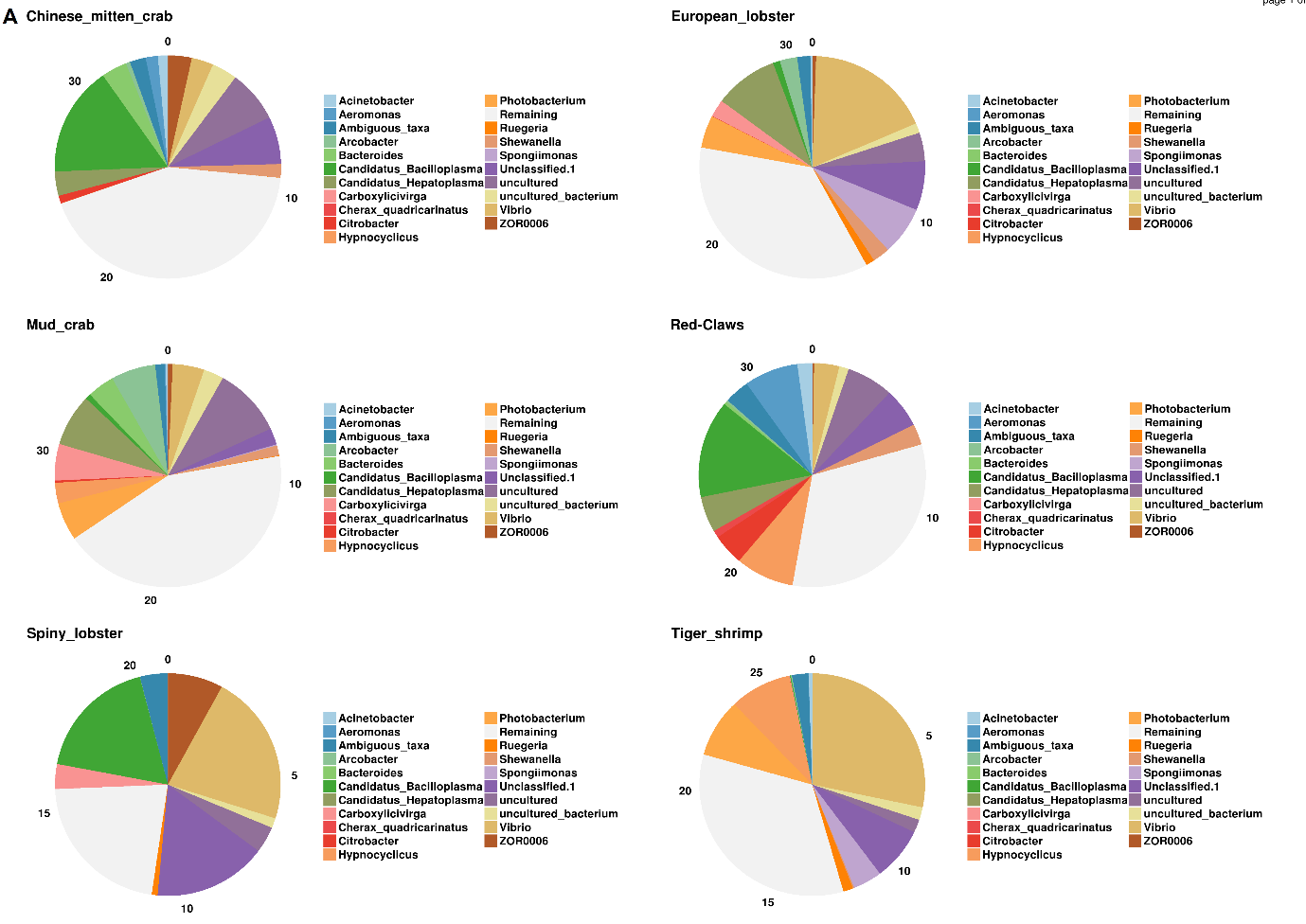


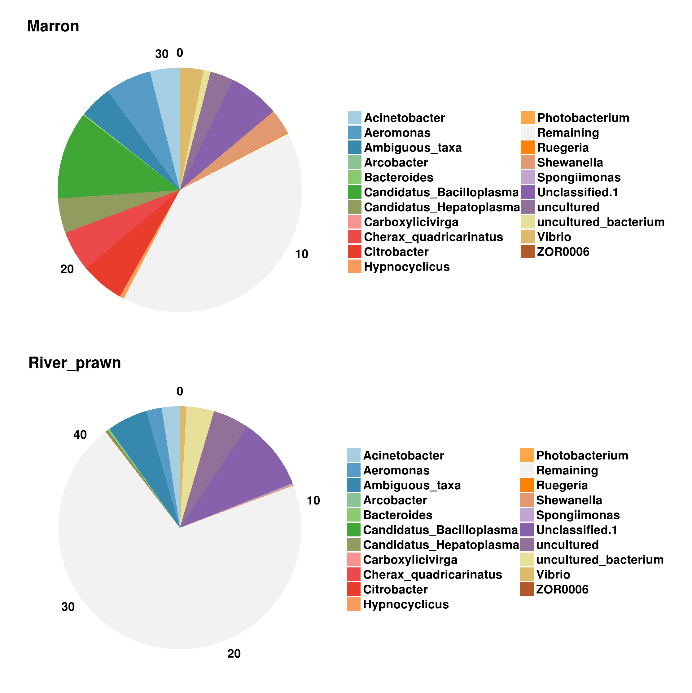

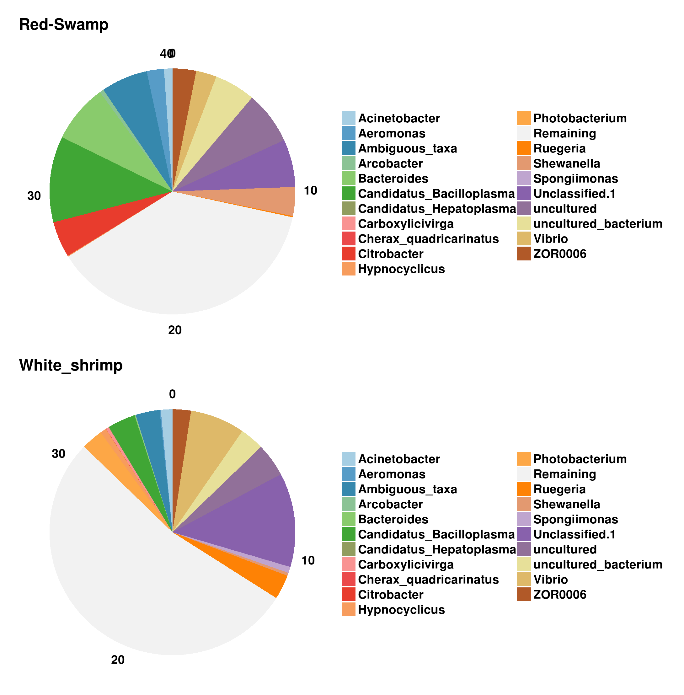

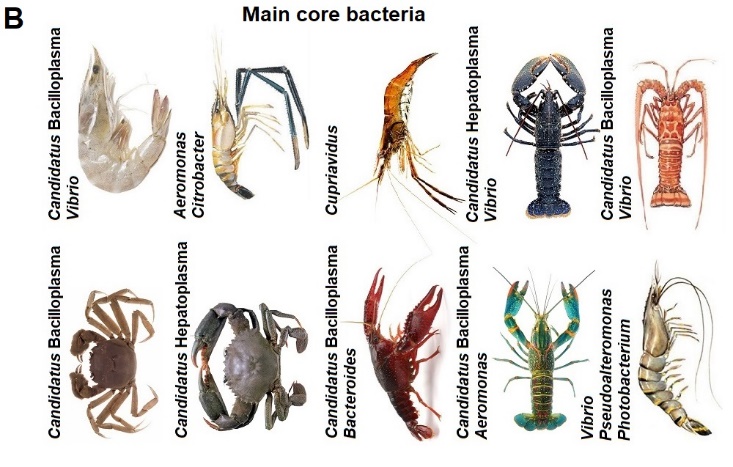

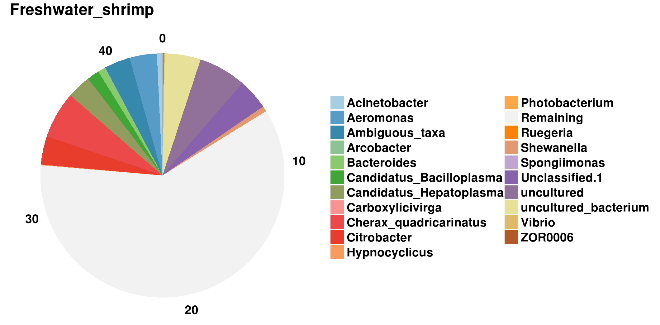


**Figure S6**. Gut microbial composition in 11 decapod crustacean species. (A) Pie-chart showing top abundant bacteria at the phylum level. (B) Core-gut bacteria in 11 decapod crustacean’s species identified in ≥95% of samples, regardless of diets, growth, health, developmental stages and environmental conditions.

Table S1. Beta-dispersion (unweighted) of DC gut microbiota

|  | Crab | Crayfish | Lobster | Prawn | Shrimp |
| --- | --- | --- | --- | --- | --- |
| Crab |  | 0.0022 | 0.0002 | 0.0212 | 0.0111 |
| Crayfish | 0.0022 |  | 0.0001 | 0.0011 | 0.0001 |
| Lobster | 0.0002 | 0.0001 |  | 0.0001 | 0.0002 |
| Prawn | 0.0212 | 0.0011 | 0.0001 |  | 0.0487 |
| Shrimp | 0.0111 | 0.0001 | 0.0002 | 0.0487 |  |

Pair-wise PERMANOVA (Panodis) p-value among five different groups

Table S2. Beta-dispersion (weighted) of DC gut microbiota

|  | Crab | Crayfish | Lobster | Prawn | Shrimp |
| --- | --- | --- | --- | --- | --- |
| Crab |  | 0.0045 | 0.0001 | 0.0511 | 0.0242 |
| Crayfish | 0.0045 |  | 0.0001 | 0.0784 | 0.0002 |
| Lobster | 0.0002 | 0.0001 |  | 0.0003 | 0.0024 |
| Prawn | 0.0511 | 0.0784 | 0.0003 |  | 0.0444 |
| Shrimp | 0.0242 | 0.0002 | 0.0024 | 0.0444 |  |

Pair-wise PERMANOVA (Panodis) p-value among five different groups

Table S3. Fisher presence-absence test of main core gut microbiota in DC

| **Genera** | **Crab** | **Crayfish** | **Lobster** | **Prawn** | **Shrimp** |
| --- | --- | --- | --- | --- | --- |
| Vibrio | 72.6%  CMC-46.5%  MC-74.6% | 64.7%  MR-72.4%  RC-63.4%  RS-68.6% | 100%  EL-100%  SL-100% | 36.2%  FP- 28.9%  RP-42.4% | 99.6%  TS-100%  WS-99.2% |
| Candidatus Bacilloplasma | 99.2%  CMC-100%  MC-98.6% | 98.6%  MR-99.8%  RC-98.2%  RS-97.4% | 52.2%  EL-14.6%  SL-78.2% | 50.7%  FP-22.4%  RP-78.9% | 41.9%  TS-8.8%  WS-72.4 |
| Aeromonas | 26.2%  CMC-42.2%  MC-18.6% | 68.5%  MR-78.2%  RC-84.6%  RS-28.4% | 12.8%  EL-18.6%  SL-12.4% | 78.9%  FP-90.6%  RP-16.4% | 22.4%  TS-8.4%  WS-24.8% |
| Candidatus Hepatoplasma | 82.8%  CMC-32.1%  MC-88.9% | 62.3%  MR-68.4%  RC-58.1%  RS-24.4% | 78.5%  EL-82.4%  SL-32.2% | 52.4%  FP-68.8%  RP-28.9% | 12.6%  TS-8.4%  WS-14.2% |
| Bacteroides | 85.6%  CMC-82.1%  MC-86.8% | 62.4%  MR-9.2%, RC-12.6% RS-100% | 4.8%  EL-4.6%  SL-8.2% | 36.6%  FP-48.2%  RP-18.9% | 3.2%  TS-2.4%  WS-4.2% |
| Hypnocyclicus | 52.7%  CMC-32.1%  MC-78.9% | 82.9%  MR-59.2%, RC-98.6% RS-54.4% | 2.2%  EL-1.9%  SL-2.4% | 3.6%  FP-4.2%  RP-2.9% | 2.1%  TS-2.1%  WS-2.9% |
| Pseudoalteromonas | 0.8%  CMC-0.5%  MC-0.9% | 0 | 6.8%  EL-7.9%  SL-3.4% | 0 | 88.9%  TS-98.6%  WS-82.4% |
| Citrobacter | 38.8%  CMC-36.5%  MC-41.9% | 65.3%  MR-62.4%  RC-84.1%  RS-44.4% | 1.2%  EL-1.1%  SL-1.3% | 72.6%  FP-92.6%  RP-22.4% | 12.1%  TS-14.2%  WS-10.8% |
| Cupriavidus | 12.8%  CMC-9.5%  MC-18.9% | 0 | 2.2%  EL-2.1%  SL-2.3% | 72.6%  FP-26.6%  RP-89.8% | 1.1%  TS-1.1%  WS-0.9% |

Percentage in the total samples counted from below equation. Abbreviations: CMC, Chinese mitten crab; MC, mud crab; MR, marron; RC, red claw; RS, red swamp; EL, European lobster; SL, spiney lobster; FP, freshwater prawn; RP, river prawn; TS, tiger shrimp; WS, white shrimp.

The percentage calculated from OTU table following taxonomy assignment with the following equation.

Genus = COUNTIF (A1:B2,">"&0)/C*100

A1:B2 = Count range for selected DC group

C = Total number of samples
